# Foreign Summary

**Published:** 1839-07-06

**Authors:** 


### FOREIGN SUMMARY.

Dr. Conolly, one of the Editors of the British and Foreign Medical Review, and author of a Treatise on Insanity, has been appointed to the important office of Resident Physician at the Hanwell Lunatic Asylum, Middlesex, near London.

---

*On the Treatment of the Purulent Ophthalmia of New-Born Infants.* By Mr. Carmichael.—The treatment should vary with the state of the disease, which we may therefore divide into two stages; the first, inflammation of the lids, purulency, and probably inflamed sclerotica; the second, when it has passed on and injured the cornea, which may be either in a state of suppuration or of ulceration, the ulcer still containing the originally deposited lymphy matter.

Mr. Saunders has very properly recommended the use of leeches in the first or inflammatory stage, and, if the inflammation be high, they should not be neglected; the great danger to be apprehended is, the cornea becoming engaged, which they mainly assist in preventing, or if this have already taken place, they often cause an absorption of the deposited lymph, and prevent the formation of an ulcer; in their use, however, we must recollect the great vascularity of the skin in infants, and the difficulty sometimes in stopping the hæmorrhage from their bites; I think two leeches, one to each eye, will be in most instances sufficient, except under very urgent circumstances, having recourse to them again if required; I prefer this to the application of a greater number at once, having seen cases where there was reason to regret the latter practice.

After what I have said as to the connexion which may exist between the bowels and the disease, it is unnecessary to say, that I consider the strictest attention to the former indispensable. The hydrargyrum cum cretâ in grain or half grain doses, twice or thrice a day, will probably be the best medicine we can administer. This, however, should be persevered in steadily, till the uneasiness and irritation of the bowels be removed, and the appearance of the dejections become natural; and of such importance do I consider this part of the treatment, that in bad, obstinate cases, I direct changing if possible the nurse, the milk of one woman often disagreeing with the child, and causing all this bowel derangement, and which is at once obviated by that of another: almond milk I have often likewise given with benefit where it was obstinate; if this be used, however, it should always be made fresh, from the tendency it has to become sour, and if the child be spoon-fed the utmost care should be taken that only so much food shall be made at a time as shall be used, and the vessel employed for that purpose be then instantly cleansed; this is a want of attention on the part of those who spoon-feed infants, which is very much to be censured; bread and milk is the pap usually given to them, and what is left from one feeding is generally kept over, and given re-warmed at the next. Now there is nothing that has a greater tendency to get rapidly sour than this kind of food; what remains from one meal is acid at the next, and its admixture with fresh food vitiates the entire. In consequence of this and of the neglect of cleaning the vessel in which it is made, we generally find fed infants, what are termed cross children, the victims of griping and infantile colic; the bread should be of the very best quality, first washed, the milk composed of equal or two parts of water, and one of milk, and sometimes a little calcined magnesia mixed through it. In very obstinate cases, I have often given, with much benefit, four grains of calomel and four drops of tincture of opium, into four or six papers, one to be taken night and morning.

I prefer mild collyria, a grain of nitrate of silver to the ounce of distilled water, or the alum wash in the proportion of two or three grains to the same complement, or acetate of lead or zinc wash, in the one grain proportion, with strict attention to the bowels in the manner stated; I think we shall scarcely fail with collyria of these strengths, in effecting any advantage to be derived from them: latterly very strong and irritating applications have been advised, in the shape of ointments or nearly saturated solutions of nitrate of silver, and from the very respectable authority they come to us recommended by, we cannot, I think, question their great utility and power in suppressing the discharge, and, perhaps, arresting the disease; for myself, however, finding as I do, how readily it yields to the mild descriptions I have mentioned, with the collateral treatment spoken of, I have continued to use them, and cannot, therefore, speak of the others from experience. I have very frequently found, that one of the above collyria will answer exceedingly well for a time, and afterwards cease in its effects, and then by changing it for any of the others, a rapid improvement takes place, or perhaps the discharge disappears altogether; change of air, I have constantly seen to act with great benefit, particularly in hospital practice; the purulency copious, while the child was in the house resisting every thing, and with the disease rapidly disappearing on leaving it.

Blisters behind the ears by some practitioners are not approved of, from the apprehension of their producing a description of ulceration very difficult of cure at this period of life; I have never, however, seen any untoward consequences from their use. Were the surface extensively broken by them, such consequences might probably ensue; the manner I employ them in, is by greasing two or three threads of worsted with the blistering ointment, and inserting the threads so greased close in behind the ears; in this way I have never seen any unpleasant description of ulceration induced, but have found their application of much benefit.

The lids are very much disposed to adhere during sleep, and their separation afterwards is productive of much irritation to the already irritated parts; it is, therefore, of consequence to prevent this from taking place, and which is readily accomplished by the use of the mild citron oitment, the unguentum nitratis hydrargyri mitius, and zinc ointment, mixed well together, a small portion of which is to be melted and inserted between them twice a day.

By these means, when the inflammation is confined to the first stage we shall mostly succeed in effecting a cure; attention to the bowels, collyria, blisters, if necessary, behind the ears, obviating the tendency of the lids to adhere, and resorting to leeches, as they may be required to keep down or subdue inflammation: the eyes themselves, however, and particularly the cornea, should be narrowly watched; but indeed, I think I may say, as regards this, which I call the first stage of it, when the direase is confined to that stage, the treatment required is but simple, and almost always efficacious; the chief thing to be attended to is the keeping down of the inflammation, whereas the attention of most practitioners, it would appear, is directed to the suppression of the discharge alone, to remove which, it is thought, is to cure the disease, and against which opinion an exception cannot be too strongly urged. The danger consists in the inflammation being transferred to the cornea, and when this does happen, a train of morbid symptoms set in, in most instances incapable of being restrained, and only to be managed when they are manageable by proper and just notions of their nature.

We have heretofore been considering the treatment when it is applicable to the first stage alone; we shall now proceed to that where the cornea becomes engaged, requiring much more judicious management, and a more experienced acquaintance with the disease. I have mentioned that this may present itself in two different states—suppuration, where the surface of the membrane is not yet extensively broken—and when ulceration has taken place; and in these two the treatment will vary.

In the first, if the deposition be extensive, the prospect is very unpromising; the probability is, it will come forward, break up the membrane deeply and extensively, and disorganization follow. Our object, however, is to induce absorption beforehand, if we be in time, and this is to be affected by decisive means only. For that purpose our reliance must chiefly be on leeches, and considering the importance of the organ at stake, they should be used with more freedom, so as to make an impression on the system of the little patient, the inflammation is at once and effectually to be extinguished, a few hours in this state generally rendering the case hopeless. Tartar emetic in very small does, the sixth or eighth of a grain three times a day, I have also used with much benefit, though I do not approve of a repeated use of it in infants generally. Blisters, in the way spoken of, are indispensable behind the ears, and the collyrium if necessary must be of the mildest description, as the alum wash. I consider the strong stimulating applications above alluded to, to be destructive in such cases; the cold alum curd applied over the lids, I think, I have seen sometimes beneficial.

If we be fortunate enough to see the case in sufficient time, we may by such means, actively resorted to, occasionally prevent the further advance of the inflammation and save the eye, as I have known it to happen in some seemingly unpromising cases. Sometimes it will clear, and on other occasions a stain will remain, nebulous only, or perhaps deeper, and forming an albugo or pearl, as it is termed; but should it not take this fortunate turn, it progresses to the next stage, when breach of the corneal texture takes place; this is what is termed slough. I have already, however, stated my objection to that term, nevertheless I shall for the present retain it for conveniency, to designate the morbid appearance it is applied to, as I shall have occasion to refer to it often.

Although where this stage of it occurs, there is an abatement of the inflammation generally, still it is in some way as yet present in a kind of sub-acute state, and while it is so, healthy action cannot set in decidedly. If the consecutive sloughing of Mr. Saunders be correct, it is a clear proof of this, as it can only result from it. I therefore, even here, would have recourse to a leech or two to subdue it; blistering or cold application I should not think of much utility.

By this time the disease may have been of long standing, and this, together with the sufferings of the infant, generally reduce it much; we should therefore be very guarded as to the extent we carry antiphlogistic means, as they must add to this already existing evil. Mr. Saunders has very judiciously advised the tonic plan to be now adopted; his recommendation of the extract of bark is also most judicious; I have given it frequently, and with the most decided benefit in the manner he recommends it, to the extent of sixteen or eighteen grains in the day in repeated doses; it must, as he states, be softened down and given in a little pap.

Sulphate of quinine, it might be supposed, from its condensing the virtues of the bark in a small space, would be peculiarly adapted for these cases. I have tried it, and in my hands it has not been as serviceable as the other preparation: the sulphate of quinine, even in the adult, often disagrees with the stomach, and if it should have that effect with the infant, which is a very likely thing, it is impossible any advance shall be made in the cure; when the stomach is deranged, local or even constitutional disease progresses but badly towards amendment.

Doctor Mackintosh has advised the exhibition of two drops of brandy to the infant in a little food. I have given it, or what is the same, good whiskey, and with very decided benefit; three or four drops is the most I have gone to, three or four times a day: the amendment in the eye is evident in a day or two, the transparent bluish tinge, spoken of in the last section, appearing; but it is in the general appearance, and particularly in the face of the child, it is manifested; the sunken, worn-out aspect giving way, and becoming replaced by the ruddy tinge of health; the bowels must still be attended to.

I do not approve of collyria in these cases; they have been advised in order that they may effect the removal of the sloughs, for the purpose, as it is said, of enabling the proper application to be made to the ulcer to stimulate it to heal; my objection is to this very point; it is on mechanical principles, and I shall state it.

We know not to what depth the slough may have gone; it may have bared the membrane of the aqueous humour extensively, and if so, its presence alone keeps the part so deduded *in situ;* its premature removal therefore must necessarily be followed by an advance of that membrane, most probably accompanied with a portion of the iris, and of course leading to the destruction of the eye. I prefer attacking the breach by the above constitutional measures, and for the present should be rather desirous for the continuance of the slough. The state of the parts probably is—deep ulcer of the cornea occupied with the slough; now, ulcers of the cornea, like those in other parts, heal from the bottom, and if we succeed in forwarding this process to a certain extent before the slough is removed, the part is, as it were, secured against the impending danger, (hernia of the iris,) and then on its removal a manageable ulcer is what we have to deal with; but under the other circumstance, the untoward result I have mentioned must follow.

It may be imagined by those who have not seen this morbid condition of the parts, that a slough could scarcely restrain the pressure from within in the way stated; more particularly if the morbid matter be diseased lymph, such as I have described it. It is, however, of peculiar tenacity; in the adult, something similar to it occasionally does take place, completely perforating the cornea, and by its remaining in its position blocks up for the time being the perforation so made. I have in such cases (for the experiment could not in the infant obviously be made) sometimes passed a fine Anel's probe completely into the anterior chamber till its point appeared behind the cornea, demonstrating in these cases the extreme tenacity of such matter, and its consequent capability to block up these openings; there is also in Mr. Saunders something tantamount to a similar statement; he observes, that the membrane of the aqueous humour is sometimes bared, with its destructive consequences, when by a succession *of sloughs* the entire of the cornea anterior to it is removed. We differ only as to the nature of the so consecutively removed parts, whether they be actually portions of cornea dead and cast off in the shape of slough, or what I have stated them to be; my reason for adopting the latter opinion I have already advanced.

Whether, however, they be sloughs, or what I contend for, is immaterial, I think, for my position; it is admitted on all hands, that advance of the iris is endangered on their complete removal, and therefore I would object to the practice that must lead to it prematurely.

For these reasons then, I am opposed to collyria in such a state of parts, and my reasons are, lest they might effect that for which they are employed by those who advise them, namely, the removal of the sloughs.

If the case goes on favourably, I mean so that the eye shall be ultimately saved, it sometimes happens that there will be a cessation of the reparative process; it will be at a stand, and resist the collyria, and all tonic means. I prefer, in such cases, throwing a jet of a one-grained solution of nitrate of silver on the ulcer from an Anel's syringe, to increasing the strength of the collyrium; if a favourable opportunity be taken while the infant sleeps, this can readily be done, and it generally excites a return of the healing process. I would again, however, observe, that we are not to be too sanguine in these cases, particularly if the lesion be at the edge of the cornea, even when matters appear favourable.

The advance of the iris is easily detected; its appearances have been already stated; it is generally the completion of the destructive process, and our object should now be to restrain it in its progress, so as if possible to save the pupil. I believe, for this purpose, there is but one means, namely, the application of the solid nitrate of silver; but in order to this, the application should be made with particular caution; a pencil of it must be thinned and brought to a very fine point, by repeatedly dipping it in water, and with a piece so prepared, the top of the little button of the iris is alone to be touched. If the application be made with rudeness, so that the caustic is brought in contact with the cornea, great irritation will be produced, and perhaps the state of parts rendered worse by it; besides, if we succeed in stopping the advance, the cure will be attended with opacity of the cornea, rendering the eye useless; but by proceeding in the way here advise, (touching solely the top of the eminence,) there is no inconvenience experienced. Very frequently, when the protrusion is not extensive, by such cautious mode of proceeding it recedes, and vision is preserved. In order to succeed in this way, a proper opportunity should be selected; when the child sleeps, the upper lid is to be then cautiously opened by the practitioner, standing at the head of the infant, and the nitrate carefully brought into contact with the part. I have tried solution of the salt in the strongest possible form, but it had little or no benefit, the solid nitrate is alone decidedly serviceable, when service can be rendered, and the caution above advised in its employment is to be attended to.

It is almost needless, however, to remark, that we cannot by any means generally expect this stage of the disease to assume so manageable a form; when protrusion takes place, the eye, in the great majority of instances, is lost, the results as to appearances being various. Most frequently, the advance of the iris, or of the iris covered with a plate of the cornea, goes on, terminately in staphyloma, which sometimes continues to extend, until the enlarged globe becomes incapable of being received within the closed lids. The deformity now is considerable; the protruded diseased cornea being constantly exposed to the action of the atmosphere, inflammation is quickly set up in it, accompanied with the greatest irritation, and only to be relieved by surgical interference, so as permanently to evacuate or destroy the globe, and thus to allow it to shrink within the orbit. I have sometimes known this state to be, to a certain extent, repressed by the action of the solid nitrate of silver, but generally after some time it ceased to have its effects, and the disease advanced; however, as this condition of the parts, when it occurs, constitutes another affection, (staphyloma,) to enter upon it or its treatment, in anything of detail, would be foreign to the object of this communication, which professes alone to speak of the purulent ophthalmia of infants.—*Dublin Journal, for May.*

---

*Glanders in the Human Subject.*—An experiment, recently performed by M. Nonat and M. Bouley, has demonstrated the identity of glanders occurring in the human subject with the glanders which affects horses. A horse was inoculated with some purulent matter taken from a patient labouring under glanders. The animal soon presented all the well-known symptoms of glanders, and died on the 11th of March, eighteen days after the inoculation. On comparing the lesions of the nasal cavity which were observed in the inoculated animal with the parts of a horse which had died of glanders occurring in the ordinary way, the points of resemblance were found to be most striking.—*Lancet, from Bul. de l'Acad. Roy. de Med.*, May, 1839.

---

*Contamination of the Fœtus by Venereal Poison.*—Dr. Kennedy lately exhibited to the Dublin Pathological Society a fœtus with discoloration of the skin and separation of the cuticle, whose mother was syphilitic. He begged also to lay before the Society the body of an infant whose mother had venereal condylomata. The skin, particularly about the buttocks, was much discoloured. The disease subsequently spread to the hams and thighs; and the child died in about six weeks after birth.

He likewise exhibited the body of another child whose mother had secondary symptoms. Her husband had died of syphilitic laryngitis. It was born with desquamation of the cuticle of the hands and feet; but soon lost all the characters of the disease.—*Dub. Journal.*

